# Supplementary material for: Loss to Follow-Up from HIV Pre-Exposure Prophylaxis Care in Men Who Have Sex with Men in West Africa
Source: Viruses. 2022 Oct 28;14(11):2380. doi: 10.3390/v14112380 (PMC9695325; doi:10.3390/v14112380)
Supplement: Supplementary file 1 [file viruses-14-02380-s001.zip › viruses-1953612-supplementary.pdf]

Supplemental Material 1

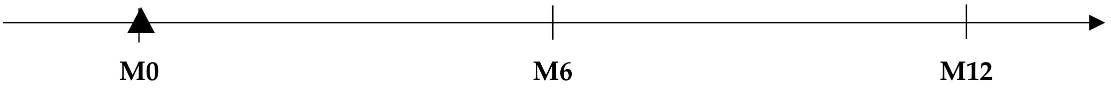

Participant 1 never returned for follow-up after their first visit. They are considered as LTFU.

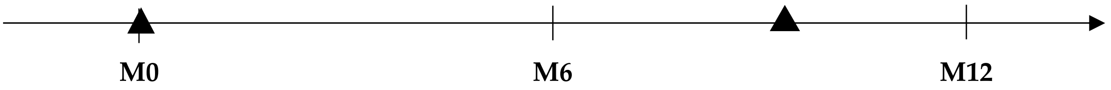

Participant 2 returned for follow-up more than 6 months after their last visit. They are considered as LTFU.

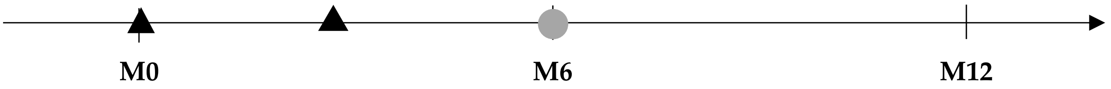

Participant 3 withdrew from care after their last visit. They are considered as LTFU.

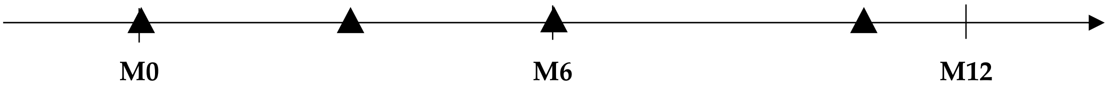

Participant 4 returned for follow-up less than 6 months after their last visit. They are not considered as LTFU.

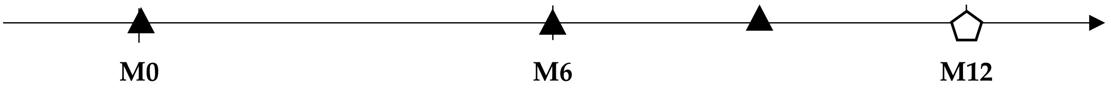

Participant 5 experienced an HIV seroconversion after their last visit. They are not considered as LTFU.

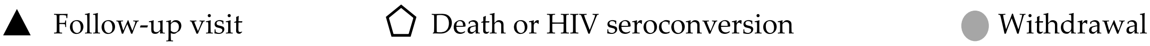

Scheme S1. Definition of LTFU
